# Detection of gastrointestinal parasitism at recreational canine sites in the USA: the DOGPARCS study

**DOI:** 10.1186/s13071-020-04147-6

**Published:** 2020-06-01

**Authors:** Kristina Stafford, Todd M. Kollasch, Kathryn T. Duncan, Stephanie Horr, Troy Goddu, Christine Heinz-Loomer, Anthony J. Rumschlag, William G. Ryan, Sarah Sweet, Susan E. Little

**Affiliations:** 1 Elanco Animal Health, 2500 Innovation Way, Greenfield, IN 46140 USA; 2 Department of Veterinary Pathobiology, Center for Veterinary Health Sciences, Oklahoma State University, Stillwater, OK 74078 USA; 3 IDEXX, 1 IDEXX Dr, Westbrook, ME 04092 USA; 4 Ryan Mitchell Associates LLC, 16 Stoneleigh Park, Westfield, NJ USA

**Keywords:** *Ancylostoma*, Centrifugal flotation, Coproantigen, Dog, *Giardia*, Hookworm, Intestinal parasite, Roundworm, *Toxocara*, *Trichuris*, Whipworm

## Abstract

**Background:**

The rapid growth in off-leash dog parks provides opportunity for canine socialization activities but carries risk of exposure to intestinal parasites. This study assessed the prevalence of these infections in dogs visiting off-leash dog parks.

**Methods:**

Fresh defecations were collected from dogs visiting parks in 30 metropolitan areas across the USA. Samples were analyzed by coproantigen immunoassay (CAI) (Fecal Dx® and *Giardia* Test, IDEXX Laboratories, Inc.) and zinc sulfate centrifugal flotation (CF). Owners responded to a questionnaire on their dog’s signalment and use of heartworm/intestinal parasite control medications (HWCM).

**Results:**

Samples were examined from 3006 dogs, 87.9% aged at least 12 months, visiting 288 parks. At least one intestinal parasite was detected in 622 (20.7%) samples, nematodes in 263 (8.8%), with hookworms, whipworms and ascarids in 7.1, 1.9 and 0.6% of samples, respectively. A sample positive for one or more intestinal parasites was found in 245 (85.1%) parks, with nematodes found in 143 (49.7%). Combined, CAI and CF detected 78.4% more intestinal nematode infections than CF alone. Hookworm and whipworm infections were detected in all age groups, but ascarids were only detected in dogs less than 4 years-old. Approximately 42% of dogs aged less than 1 year were positive for nematodes or *Giardia*. Based on owner reports, HWCM was current for 68.8% of dogs, dogs previously diagnosed with intestinal parasitism were more likely to be receiving a HWCM than those without such history, and a significantly lower (*P* = 0.0003) proportion of dogs receiving a HWCM were positive for intestinal nematodes compared with those not on such medication.

**Conclusions:**

Intestinal parasites, the most common of which were *Giardia*, *Ancylostoma caninum* and *Trichuris vulpis*, were found in 20% of dogs and 85% of dog parks across the USA. Enhanced detection of canine intestinal parasitism was achieved by combining CF and CAI. Canine intestinal parasites are common across the USA and dog health can be improved by regular testing of fecal samples and routine administration of medications effective against the most common infections.
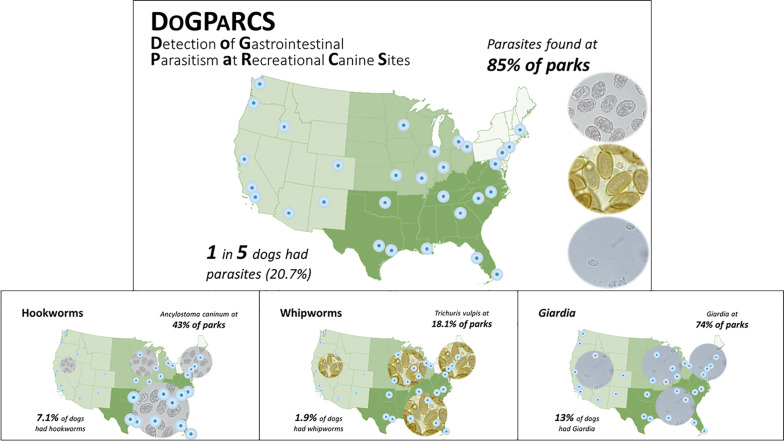

## Background

Canine intestinal parasite infections are often subclinical but can become clinically apparent in puppies and in adults with heavy burdens [1, 2]. Subclinical infections may carry a health cost, and dogs with patent infections shed eggs, oocysts or cysts that can contaminate the environment and act as a source of reinfection, infection of other dogs, and in some cases infection of humans [3, 4]. Stray and shelter dogs have higher rates of patent infections than dogs in the general population and are often rescued and relocated across country and state borders [2, 5–7].

Humane organizations care for and relocate shelter dogs, helping to address the demand for new pets, with pet dog ownership in the USA now the highest since measuring began in 1982 [8]. If newly-homed dogs are harboring intestinal parasites and left untreated, they can be a source of infection for other dogs in their new locations. Additionally, the relationship between dogs and owners has continued to evolve with more people incorporating their dogs into daily activities and travel than in previous generations, exposing the dog to environments potentially frequented by many other dogs [8–10]. Understanding the national risk of canine intestinal parasite infection is therefore important to drive recognition of the need for effective testing and control measures.

Two published reports have provided insights into the national prevalence of intestinal parasite infection of dogs in the USA [2, 5]. In the first report, findings were based on centrifugal sucrose flotation of fecal samples collected from shelter dogs, while in the second the results were based on samples submitted by veterinary practices to a testing laboratory that utilized zinc sulfate centrifugal flotation (CF). Extrapolation of results of the former study to the pet dog population is limited because shelter dogs may not have received anthelmintic treatment, and 84% of sampled dogs were under 3 years of age. Nonetheless, the findings of intestinal parasite infection in those dogs provide an indication of the potential parasite transmission risk associated with the movement of shelter dogs. A limitation of the second study is that samples were submitted by veterinary practices and so would likely have come from dogs receiving consistent high-quality veterinary care.

Both previously conducted national studies used CF, a strategy that is more sensitive than the passive flotation technique commonly used in veterinary practices. However, CF is not able to detect non-patent infections, may fail to identify low intensity infections or those in which particularly dense eggs are shed (e.g. cestodes, trematodes), and can, when high specific gravity solutions are used, collapse or fail to recover *Giardia* cysts. To address these limitations, coproantigen immunoassays (CAI) have been developed to detect proteins found in *Giardia* cysts or produced by immature and adult nematodes in the intestinal lumen [11–15]. When used with CF, CAI enhances the detection of canine intestinal parasite infections [14–18]. With the interstate relocation of dogs that could be infected with intestinal parasites and the availability of improved testing methodologies, there is opportunity for an up-to-date assessment of intestinal parasitism in dogs in the USA.

Over the last ten years across the USA, the number of off-leash dog parks has increased dramatically, providing ideal locations to sample pet dogs [19]. A national study was initiated with the objective of determining the prevalence of canine intestinal parasite infections in dogs visiting dog parks. Other objectives were to provide insight into the complementary use of CF and CAI testing to diagnose these infections, and to assess the relationship between owner-reported use of heartworm control medications (HWCM) and intestinal parasite infection.

## Methods

### Fecal samples

Dog parks were selected in 30 major metropolitan areas across the USA (Table 1). Investigators collecting samples were veterinary staff from the College of Veterinary Medicine at Oklahoma State University and veterinary staff of Elanco Animal Health and IDEXX Laboratories, Inc. For each metropolitan area, 10 parks were selected to represent the diversity of geographical, socioeconomic and neighborhood types available in the area, taking into account factors such as safety for those collecting samples and accessibility of the parks. The target was to collect 100 samples from each metropolitan area. Consistent with earlier national surveys, results were divided into four regional areas derived from a previously described segmentation [2, 5].

**Table 1:** Listing of cities (alphabetical order within region) for sample collection

Southeast	Northeast	Midwest	West
Atlanta	Boston	Chicago	Albuquerque
Austin	New York City	Cleveland	Bakersfield
Charlotte	Philadelphia	Detroit	Boise
Houston	Washington DC	Indianapolis	Denver
Miami/Ft Lauderdale		Kansas City	Los Angeles
Nashville		Minneapolis	Phoenix
New Orleans		St Louis	Portland
Oklahoma City/Tulsa			Sacramento
Raleigh/Durham			Seattle
Tampa			

All dogs from which fecal samples were collected were owned by or under the care of dog park attendees and participation was voluntary. Immediately after defecation, the dog’s fecal sample was placed into a plastic bag. No samples were collected from dogs belonging to any employees of the companies supporting the study, their friends or family members, investigators or assistant investigators, or to any staff known to be employed at a veterinary clinic. Dogs brought to the park by professional dog walkers were not eligible. For owners with multiple dogs, only one dog was sampled. The person responsible for the dog had to agree for the feces to be collected and verbally respond to a study questionnaire, which included the dog’s signalment and the questions “Is your dog currently on a heartworm/intestinal worm preventive/medication?” and “Has your dog ever been diagnosed with intestinal worms?” Owners responding positively to the former question were asked whether the heartworm/intestinal worm control medication (HWCM) was administered orally, topically or by injection. Brand names were neither asked nor recorded if the owner volunteered the name.

All samples were processed at a single laboratory (IDEXX, 401 Industry Rd, Louisville, KY 40208, USA) employing validated CAIs for hookworm, whipworm and ascarids (Fecal Dx® and *Giardia* Test, IDEXX Laboratories, Inc., Westbrook, Maine, USA) and *Giardia* [15–17]. A zinc sulfate CF (specific gravity 1.24) was also used to detect a variety of parasites, including but not limited to nematodes and protozoans [20].

### Analysis of results

The proportion of dogs testing positive for each parasite was determined according to signalment, metropolitan area and region in which the dog was sampled, whether the dog’s owner reported administering a HWCM, and if the dog had been previously diagnosed with intestinal parasites. A 2-proportion z-test was used to test whether the proportion of dogs returning positive fecal tests for hookworm, whipworm, or ascarids was lower when owners reported the use of a HWCM than when owners reported not using a HWCM. A 2-proportion z-test was also used to test whether dogs reporting a previous intestinal parasite infection were more likely to be currently receiving a HWCM than those without a previous infection.

Dogs were categorized by age grouping in alignment with recent American Animal Hospital Association guidelines, with consistent years applied to each category: puppy, < 1 year-old; young adult, 1 to 3 years-old; mature adult, 4 to 6 years-old; and senior, ≥ 7 years-old [21]. A 4-sample test for equality of proportions was used to test whether the proportion of dogs testing positive for hookworm, whipworm, ascarid or *Giardia* varied between these age groups.

Holm’s multiple comparison correction was used to control the family-wise error rate due to the large number of comparisons being made. *Post-hoc* pairwise comparisons of the proportion of HWCM usage by region were performed using Holm’s multiple comparison correction.

Throughout, 95% confidence intervals (CI) were calculated using the modified Wald method, except for the use of HWCMs by age and by region for which a multinomial approach was employed to model the individual probabilities of the response variable (age category × preventive) [22].

## Results

### Demographics and questionnaire

Samples were collected from 3022 dogs in 288 dog parks during July and August 2019. Sixteen samples were disqualified because they were not accompanied by completed questionnaires or because insufficient feces were available for testing. Thus, fecal testing results and questionnaires were available from 3006 dogs. The most commonly represented age group was young adult (1 to 3 years-old) (*n* = 1371, 45.6% of 3006 dogs), followed by senior (≥ 7 years-old) (659, 21.9%), mature adult (4–6 years-old) (613, 20.4%) and puppy (< 12 months) (363, 12.1%). Within the puppy group, 72 dogs (2.4%) were less than 6 months of age. Of the 3006 dogs, 1317 (43.8%) were female, of which 1183 (89.8%) had been spayed while 1689 (56.2%) were male, of which 84.6% had been neutered. As reported by owners, the most commonly represented breeds were: Labrador Retriever (356, 11.8% of sampled dogs), German Shepherd Dog (187, 6.2%), Golden Retriever (137, 4.6%), Australian Shepherd (103, 3.4%), Siberian Husky (102, 3.4%), Chihuahua (83, 2.8%) and Boxer (64, 2.1%). The breeds of 547 dogs (18.2%) were described as mixed or were not specified.

In response to the question on nematode parasite control, 2069 owners (68.8%) stated that they were currently providing a HWCM for their dog. Of those owners, 1847 (89.3%) reported using an oral formulation, 68 (3.3%) a topical formulation and 144 (7.0%) an injection. Ten owners did not know how the HWCM was being administered (Table 2). The proportion of dogs reported to be currently receiving a HWCM was significantly higher (*P* < 0.0001) in those previously infected with intestinal parasites (79.2%) (estimated difference of proportions 0.111; 95% CI: 0.075–1), compared with those without known prior intestinal worm infection (68.1%). Regionally, owner-reported HWCM use in the West was significantly lower than in each other region (*P* < 0.0001). No other between-region differences were significant. By dog age grouping, the frequency of use of a HWCM was similar at approximately 70% for puppies through mature adult dogs, while 63.6% of owners of senior dogs reported that they were providing a HWCM.

**Table 2:** Number of owners (%) reporting currently using a heartworm/intestinal parasite control medication, by region and formulation

	Southeast (*n* = 989)	Northeast (*n* = 400)	Midwest (*n* = 708)	West (*n* = 909)	National ( *N* = 3006)
Medication use					
Yes	829 (83.8)	317 (79.3)	570 (80.5)	353 (38.8)	2069 (68.8)
95% CI	81.5–86.1	75.3–83.2	77.6–83.4	35.7–42.0	67.2–70.5
No	14 (14.8)	68 (17.0)	135 (19.1)	536 (59.0)	885 (29.4)
95% CI	12.6–17.0	13.3–20.7	16.2–22.0	55.8–62.2	27.8–31.1
Unknown	14 (1.4)	15 (3.8)	3 (0.4)	20 (2.2)	52 (1.7)
95% CI	0.7–2.2	1.9–5.6	0.1–0.9	1.3–3.2	1.3–2.2
Formulation (*n*, % of dogs on heartworm/intestinal control medication)					
Oral	728 (87.8)	299 (94.3)	497 (87.2)	323 (91.5)	1847 (89.3)
95% CI	85.6–90.0	91.8–96.9	84.5–89.9	88.6–94.4	87.9–90.6
Topical	23 (2.8)	8 (2.5)	27 (4.7)	10 (2.8)	68 (3.3)
95% CI	1.7–3.9	0.8–4.3	3.0–6.5	1.1–4.6	2.5–4.1
Injectable	74 (8.9)	9 (2.8)	43 (7.5)	18 (5.1)	144 (7.0)
95% CI	7.0–10.9	1.0–4.7	5.4–9.7	2.8–7.4	5.9–8.1

*Abbreviation*: CI, confidence interval

### Fecal test results

Using CAI and CF, intestinal parasites were detected in 622 (20.7%) samples, with 8.8% positive for one or more of hookworms, whipworms and ascarids (Table 3). Of the 288 parks, 245 (85.1%) provided samples positive for any intestinal parasites, with 49.7% positive for at least one of the aforementioned nematode groups. The most commonly detected parasite was *Giardia*, while hookworm was the most commonly detected nematode group. Other canine parasites detected on CF included *Cystoisospora* spp. (*n* = 16 dogs), *Alaria* sp. (*n* = 1), capillariids (*n* = 2), *Spirometra* sp. (*n* = 2) and a taeniid egg (*n* = 1). The spurious parasites *Eimeria* spp. were identified in samples from 37 (1.2%) of dogs.

**Table 3:** Number (%) [95% CI] of dogs and parks with ≥ 1 sample positive for intestinal parasites by coproantigen immunoassay or centrifugal flotation

Parasite species	Dogs	Dog parks
	*n* (%) [95% CI] (*N* = 3006)	*n* (%) [95% CI] (*N* = 288)
Any parasitic species^a^	622 (20.7) [19.3–22.2]	245 (85.1) [80.5–88.8]
Nematodes^b^ and/or *Giardia*	609 (20.3) [18.9–21.7]	243 (84.4) [79.7–88.1]
*Giardia* spp.	391 (13.0) [11.9–14.3]	213 (74.0) [68.6–78.7]
Nematodes^b^	263 (8.8) [7.8–9.8]	143 (49.7) [43.9–55.4]
Hookworms	214 (7.1) [6.3–8.1]	125 (43.4) [37.8–49.2]
Whipworms	58 (1.9) [1.5–2.5]	52 (18.1) [14.0–22.9]
*Eimeria* spp.	37 (1.2) [0.9–1.7]	33 (11.5) [8.2–15.7]
Ascarids	17 (0.6) [0.3–0.8]	16 (5.6) [3.4–8.9]
*Cystoisospora*	16 (0.5) [0.3–0.9]	16 (5.6) [3.4–8.9]
*Alaria*	1 (0.0) [0–0.2]	1 (0.4) [0–2.1]
Capillariids	2 (0.1) [0–0.3]	1 (0.4) [0–2.1]
*Spirometra*	2 (0.1) [0–0.3]	2 (0.7) [0–2.7]
Taeniids	1 (0.0) [0–0.2]	1 (0.4) [0–2.1]

^a^Includes all parasitic species of nematodes, as well as *Alaria*, *Cystoisospora* and *Spirometra* and taeniids

^b^Nematodes: hookworms, whipworms, ascarids (includes co-infections)

*Abbreviation*: CI, confidence interval

Use of CF and microscopy allowed identification of hookworm and ascarid ova. Of 110 samples positive for hookworm ova, 108 (98.2%) were *Ancylostoma caninum* and 2 (1.8%) were *Uncinaria stenocephala*. Of 12 samples positive by CF for ascarids, 11 (91.7%) were *Toxocara canis* and 1 (8.3%) was *Toxascaris leonina*. Of the 42 ova with bipolar plugs, two were *Eucoleus aerophilus* and the remainder were *Trichuris vulpis*.

**Fig. 1:**
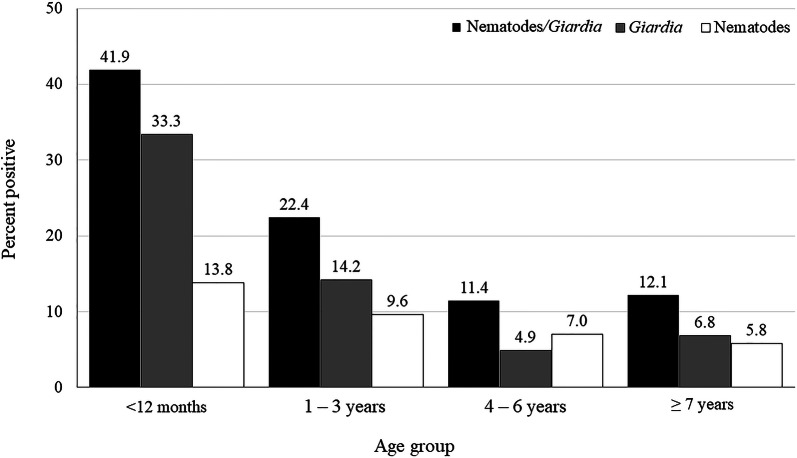
Percent of dogs in each age group positive for intestinal parasites

**Fig. 2:**
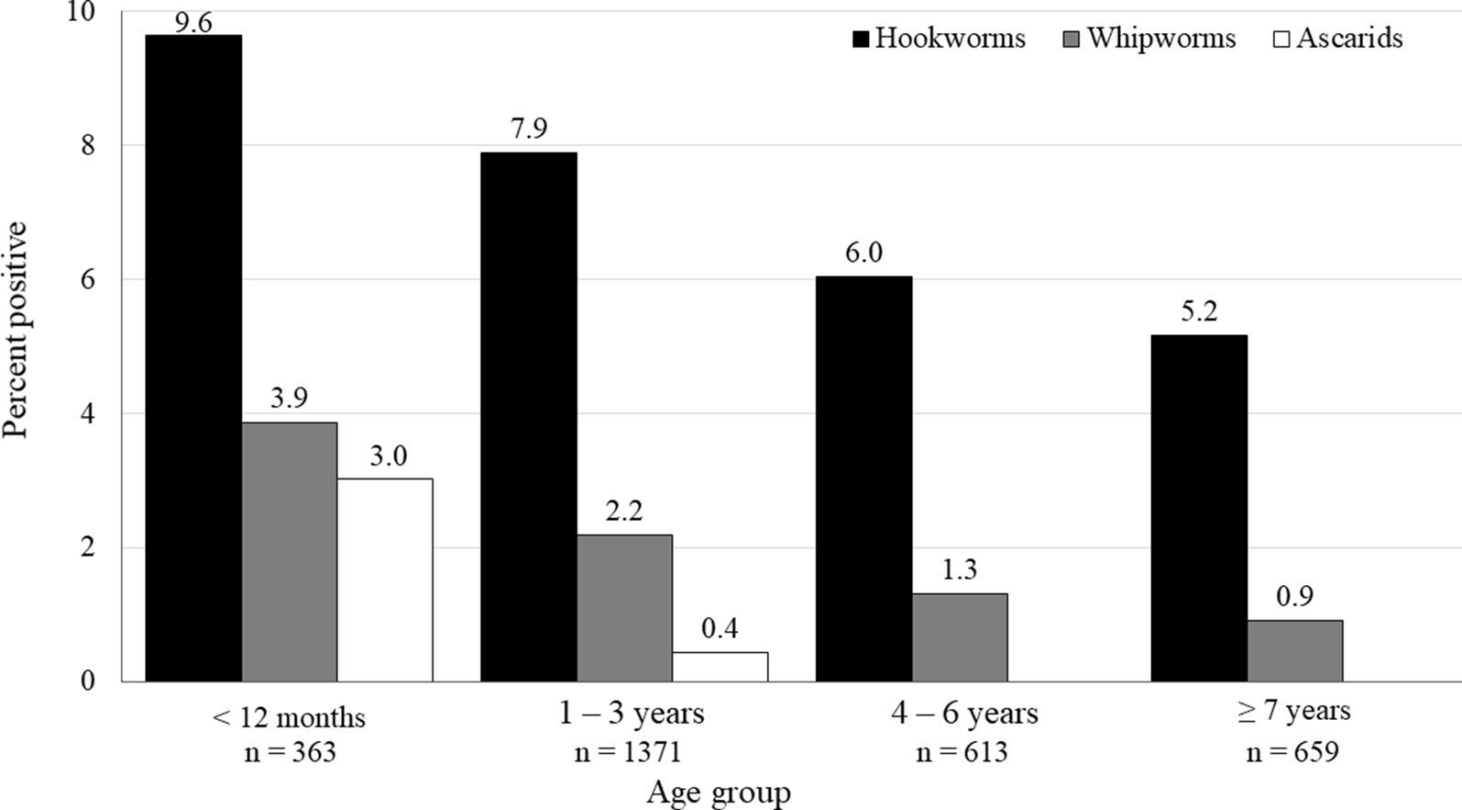
Percent of dogs in each age group positive for a nematode parasite. Ascarids were not detected in dogs < 4 years of age

A 4-sample test for equality of proportions of age groups with positive tests for one or more of hookworm, whipworm and ascarids was significant (*P* < 0.0001), with the highest prevalence in dogs less than 12 months of age. Infection with hookworm, whipworm and *Giardia* was detected regardless of age group, while ascarid infection was only identified in samples from dogs under 4 years of age (Figs. 1, 2; Table 4). Dogs reported to be currently on a HWCM had a significantly lower proportion of positive test results for hookworms, whipworms or ascarids (7.5%) than those not receiving a HWCM (11.4%) (*P* = 0.0003; estimated difference of proportions − 0.039; 95% CI: − 1 to − 0.018) (Table 5).

**Table 4:** Number (%) of dogs positive for intestinal parasites by coproantigen immunoassay or centrifugal flotation by age grouping

Demographic category	Nematodes/*Giardia*	Nematodes	Hookworms	Whipworms	Ascarids	*Giardia*
< 12 months; puppy (*n* = 363)	152 (41.9)	50 (13.8)	35 (9.6)	14 (3.9)	11 (3.0)	121 (33.3)
95% CI	36.9–47.0	10.6–17.7	7.0–13.1	2.3–6.4	1.6–5.4	28.7–38.3
1–3 years; young adult (*n* = 1371)	307 (22.4)	132 (9.6)	108 (7.9)	30 (2.2)	6 (0.4)	195 (14.2)
95% CI	20.3–24.7	8.2–11.3	6.6–9.4	1.5–3.1	0.2–1.0	12.5–6.2
4–6 years; mature adult (*n* = 613)	70 (11.4)	43 (7.0)	37 (6.0)	8 (1.3)	0 (0.0)	30 (4.9)
95% CI	9.1–14.2	5.2–9.3	4.4–8.2	0.6–2.6	0.0–0.8	3.4–6.9
≥ 7 years; senior (*n* = 659)	80 (12.1)	38 (5.8)	34 (5.2)	6 (0.9)	0 (0.0)	45 (6.8)
95% CI	9.9–14.9	4.2–7.8	3.7–7.1	0.4–2.0	0.0–0.7	5.1–9.0

*Note*: Percentages are based on the number of positive dogs in that age group as the numerator and total of dogs reported to be in that category as denominator

*Abbreviation*: CI, confidence interval

**Table 5:** Number (%) of dogs positive for intestinal parasites by coproantigen immunoassay or centrifugal flotation according to owner-reported current use of a heartworm/intestinal parasite control medication

Demographic characteristic	Nematodes/*Giardia*	Nematodes	Hookworms	Whipworms	Ascarids	*Giardia*
Dogs on medication: Yes (*n* = 2069)	404 (19.5)	155 (7.5)	125 (6.0)	31 (1.5)	11 (0.5)	276 (13.3)
95% CI	17.9–21.3	6.4–8.7	5.1–7.2	1.1–2.1	0.3–1.0	11.9–14.9
Dogs on medication: No (*n* = 885)	190 (21.5)	101 (11.4)	84 (9.5)	25 (2.8)	6 (0.7)	106 (12.0)
95% CI	18.9–24.3	9.5–13.7	7.7–11.6	1.9–4.2	0.3–1.5	10.0–14.3
Medication status unknown (*n* = 52)	15 (28.9)	7 (13.5)	5 (9.6)	2 (3.9)	0 (0.0)	9 (17.3)
95% CI	18.3–42.4	6.4–25.6	3.8–21.0	0.3–13.7	0.0–8.2	9.2–30.0

*Note*: Percentages are based on the number of positive dogs in that demographic category as the numerator and total of dogs reported to be in that category as denominator

*Abbreviation*: CI, confidence interval

Co-infections were detected in 49 (1.6%) dogs. The most common co-infection was hookworm + *Giardia* in 24 dogs (0.8%), followed by hookworm + whipworm co-infection in 12 dogs (0.4%). Prevalence was also calculated by region (Table 6). In each region, less than 1% of tests were positive for *Cystoisospora*. Details of the proportion of infected dogs and parks from which positive samples were collected in each of the 30 metropolitan areas are provided in Additional file 1: Table S1 and Additional file 2: Table S2.

**Table 6:** Regional distribution: number (%) of dogs and parks with a positive test (coproantigen immunoassay or centrifugal flotation) for intestinal parasites

Positive tests	Nematodes/*Giardia*	Nematodes	Hookworms	Whipworms	Ascarids	*Giardia*
From dogs						
Southeast (*n* = 989)	270 (27.3)	169 (17.1)	151 (15.3)	27 (2.7)	5 (0.5)	129 (13.0)
95% CI	24.6–30.2	14.9–19.6	13.2–17.7	1.9–4.0	0.2–1.2	11.1–15.3
Northeast (*n* = 400)	72 (18.0)	25 (6.3)	21 (5.3)	8 (2.0)	1 (0.3)	48 (12.0)
95% CI	14.5–22.1	4.2–9.1	3.4–7.9	1.0–4.0	0–1.6	9.2–15.6
Midwest (*n* = 708)	131 (18.5)	44 (6.2)	28 (4.0)	15 (2.1)	6 (0.9)	98 (13.8)
95% CI	15.8–21.5	4.7–8.3	2.7–5.7	1.3–3.5	0.3–1.9	11.5–16.6
West (*n* = 909)	136 (15.0)	25 (2.8)	14 (1.5)	8 (0.9)	5 (0.6)	116 (12.8)
95% CI	12.8–17.4	1.9–4.1	0.9–2.6	0.4–1.8	0.2–1.3	10.7–15.1
From parks						
Southeast (*n* = 96)	86 (89.6)	73 (76.0)	69 (71.9)	25 (26.0)	5 (5.2)	70 (72.9)
95% CI	81.7–94.4	66.6–83.5	62.1–79.9	18.3–35.7	2.0–11.9	63.2–80.8
Northeast (*n* = 39)	31 (79.5)	17 (43.6)	16 (41.0)	7 (18.0)	1 (2.6)	28 (71.8)
95% CI	64.2–89.5	29.3–59.0	27.1–56.6	8.7–33.0	0–14.4	56.1–83.6
Midwest (*n* = 68)	59 (86.8)	33 (48.5)	26 (38.2)	12 (17.7)	5 (7.4)	50 (73.5)
95% CI	76.5–93.1	37.1–60.2	27.6–50.1	10.2–28.5	2.8–16.5	61.9–82.6
West (*n* = 85)	67 (78.8)	20 (23.5)	14 (16.5)	8 (9.4)	5 (5.9)	65 (76.5)
95% CI	68.9–86.3	15.7–33.6	10.0–25.9	4.6–17.7	2.2–13.4	66.4–84.3

*Note*: See Additional file 1: Table S1 and Additional file 2: Table S2 for detailed numbers by metropolitan region

*Abbreviation*: CI, confidence interval

### Coproantigen immunoassay and centrifugal flotation

**Fig. 3:**
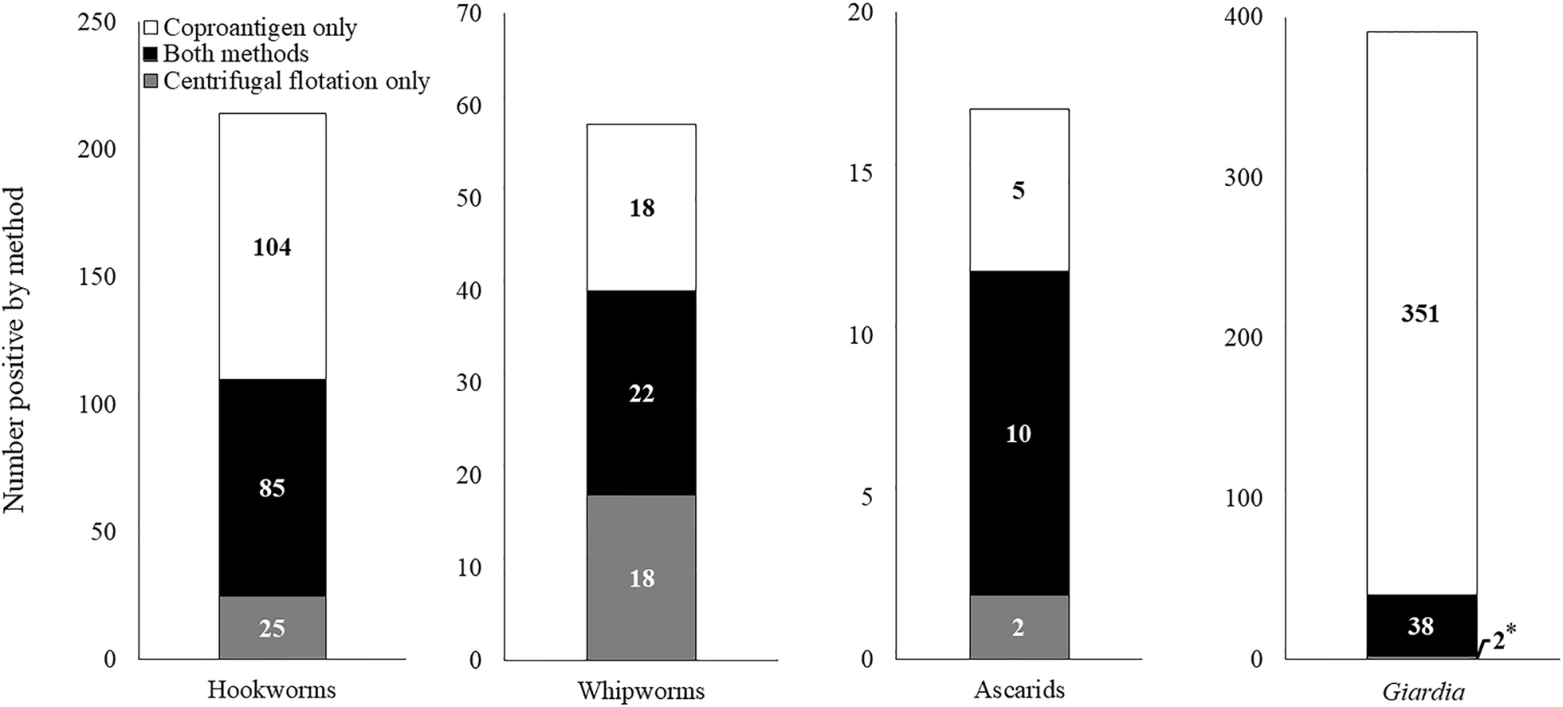
Number of infections identified by centrifugal flotation only, both methods and coproantigen immunoassay only (*two samples were positive by centrifugal flotation only)

In detection of hookworm, whipworm, or ascarids, 289 infections were found (Fig. 3). Of these, 162 (56.1%) were detected using CF and 244 (84.4%) using CAI. Both methods were in positive agreement in 117 (40.5%) of these infections. The combination of CF and CAI detected 78.4% more infections than did CF alone. For hookworm, the methods were in positive concordance for 85 infections (39.7% of detected hookworm infections), CAI detected 104 infections (48.6%) when CF was negative, and for 25 infections (11.7%) the reverse was true. Of the 58 *T. vulpis* infections, the findings for each method were in positive concordance for 22 (37.9%), 18 infections (31.0%) detected by CAI were negative on CF, and 18 (31.0%) infections detected by CF were negative by CAI. For *Giardia*, the methods were in positive concordance for 38 infections (9.7%), 351 (89.8%) infections detected by CAI were negative on CF, and 2 (0.5%) infections detected by CF were negative on CAI.

## Discussion

The present study is the first large-scale effort to determine the prevalence of intestinal parasites in dogs visiting dog parks throughout the USA. In 2019, the 100 largest USA cities contained a total of 810 dedicated dog parks [19]. Testing of samples collected from 288 parks in 30 of these cities allowed us to document parasites in over 20% of dogs and 85% of parks. The prevalence of parasite infection in dogs in the present study is higher than that seen in pet dogs with fecal samples submitted from veterinary practices to national diagnostic laboratories (12.5%), but lower than that reported from stray dogs upon arrival at municipal shelters (36%). The differences in findings between the pet dog survey and this study may be due to CAI detecting some infections missed by CF, the only method used in the earlier survey, and to the fact that while dogs attending dog parks receive attention from their owners, not all benefit from routine veterinary care [2, 5].

*Giardia* was the most commonly identified intestinal parasite, both in the present study (13.0%) and in an earlier national report of parasites in pet dogs (4.0%) [2]. In contrast, *Giardia* was only rarely detected (0.6%) in a national survey of shelter dogs in which samples were examined by sugar CF, presumably due to the lower sensitivity of this method for recovering the fragile cysts [5, 12]. Infections with *Giardia* are often subclinical, and cysts or trophozoites are shed intermittently from infected dogs, limiting the diagnostic sensitivity of CF as a stand-alone method. This may have been one factor behind the much higher rate of detection of *Giardia* infection by CAI than by CF, as would the fragility of *Giardia* cysts, leading to their degeneration between collection of fresh samples and CF testing at the laboratory. By combining CAI with CF, the present study may better estimate the true prevalence of infection. An earlier report using a similar strategy (CF + CAI) found a *Giardia* prevalence of 15.6% among dogs presenting to clinics with diarrhea or vomiting [23]. Dogs in shelters, breeding facilities and kennels are more likely to be infected with *Giardia*, and an increased prevalence among dogs that visit dog parks, compared with those not visiting dog parks, has been reported [24].

Nematodes were also commonly detected, identified in 10% of the samples tested. As in other studies using CF alone, the hookworm *A. caninum* and whipworm *T. vulpis*, which present a risk to canine health throughout all life stages of a dog, were the most common intestinal nematodes identified [14, 25, 26]. The results may underestimate the prevalence of *T. vulpis* and *T. canis*, as samples were collected during July and August, a time when infections with these nematodes may be at their lowest prevalence [27]. Surprisingly, passive flotation remains the most commonly used technique in clinical practice despite multiple studies demonstrating that it fails to detect many infections when compared to CF [12–15]. Combining CAI for nematode antigens with CF in the present study resulted in detection of nearly 80% (78.4%) more nematode infections than CF alone, likely due to the CAI detecting non-patent infections [16, 17]. Detection of parasite ova by CF in instances when CAI was negative could be due to coprophagia or predation, resulting in a positive CF in the absence of infection. In this study, 37 (1.2%) samples tested positive for *Eimeria* spp., supporting the role coprophagy may have played in the discordant results. Another factor could be that a low intensity infection may not produce sufficient antigen, leading to a negative CAI even though some ova were being shed [14, 17]. These findings reinforce the previously demonstrated complementary value of combining CAI with CF to enhance intestinal nematode detection [28].

Cestodes or trematodes were only rarely detected in the present study, even though recent studies in the USA have shown that the prevalence of infection with common tapeworms (e.g. *Dipylidium caninum*, *Taenia* spp.) is greater than that of nematodes in some populations of dogs and cats [14, 29]. Because eggs of cestodes common in dogs are shed in proglottids, and because most cestode and trematode eggs are heavy, recovery by CF is poor [30]. Using a higher specific gravity sugar solution for CF in part addresses this limitation, enhancing recovery of taeniid eggs, but sensitivity remains very low for identifying *Dipylidium caninum* infection [14, 30], and CAI is not yet commercially available for canine cestodes or trematodes. Eggs of *Spirometra* sp. or *Alaria* sp., less common cestodes and trematodes of dogs, respectively, are occasionally detected on CF. In the present study, *Spirometra* sp. eggs were identified in two dogs and *Alaria* sp. eggs in one dog.

As with earlier national reports, although parasites are found in every region, the present study indicates that the highest prevalence of nematode intestinal parasite infection, and in particular *A. caninum* infection, occurs in the Southeast [2, 5]. This factor, together with the very high prevalence of heartworm infection in the southeastern USA, likely explains the common owner-reported use of HWCM in this region. Other canine surveys from the region support that hookworm and whipworm are very common, identifying *A. caninum* in as many as 48% of shelter dogs and 17% of samples from dog parks, and *T. vulpis* in up to 39% of shelter dogs and 8.5% of samples from dog parks [14, 26, 31, 32].

In the present study, more than 15% of dogs visiting dog parks in the Southeast, and 4 to 5.3% of those in the Midwest and Northeast, were infected with hookworm, a finding that is particularly concerning given the recent reports of multiple drug-resistant hookworms in pet dogs, including Greyhounds [33–35]. Although we do not know the resistance status, six of the 12 Greyhounds sampled from dog parks in the present study were positive for hookworm, and five of those six were reported by the owner to be on a HWCM at the time they were sampled, compared to 57.7% of non-Greyhound, hookworm positive dogs that were reportedly receiving a HWCM (data not shown). Hundreds of thousands of stray and rescue dogs, including retired Greyhounds, are commonly relocated from the southern USA to other regions, a practice that can facilitate movement of parasites, including resistant parasites [7, 36, 37].

Intestinal nematodes, particularly *A. caninum* and *T. vulpis*, but not all intestinal parasites (e.g. *Giardia*, *Cystoisospora* spp.), were less commonly detected in samples from dogs reportedly receiving HWCM in the present study, providing evidence that implementing broad-spectrum parasite control measures reduces infections and limits environmental contamination with eggs. This finding has long been suspected and is supported by other regional surveys [26, 38]. However, hookworm, whipworm, or ascarid infections were still detected in some dogs reportedly receiving HWCM in the present study, perhaps due to the earlier detection afforded by antigen testing, the short (2‒3 weeks) prepatent period of hookworm, and the fact that not all HWCMs are effective against whipworm or other intestinal nematodes [16, 17, 28, 39]. For example, injectable products are not effective against either whipworm or ascarids, and are not FDA-label approved for efficacy against new hookworm infections beyond the time of initial administration [40, 41]. Products containing ivermectin/pyrantel are effective against *A. caninum*, *Ancylostoma braziliense* and *U. stenocephala*, but are not effective against whipworms [42]. Those containing milbemycin oxime are effective against whipworms, ascarids and the common hookworm, *A. caninum*, but not against its relatively scarce and less pathogenic relative, *U. stenocephala* [43]. Topically applied moxidectin is indicated to treat and control *T. vulpis*, *A. caninum*, *U. stenocephala* and ascarids [44]. The hookworm efficacy of all these treatments is based on having demonstrated efficacy prior to reports suggesting the incipient emergence of multi-drug resistant *A. caninum* [33, 35]. Detection of nematode infections and other parasites in dogs reported to be receiving HWCMs in the present study indicates that regular testing is warranted for all dogs even when these medications are used.

Interestingly, a majority (68.8%) of owners in the present study reported current use of a HWCM, similar to other recent papers surveying dog owners in Oklahoma and Florida [26, 45]. This high owner-reported prevalence of use contrasts with other data indicating that, even in areas where heartworm infection is common, only a minority of pet dogs receive a HWCM [46, 47]. Factors that may bias owner-reported use of HWCM include forgetfulness, guilt about not following veterinary recommendations, and confusion about a given product’s efficacy for heartworm *versus* external parasites. Additionally, the study was conducted in the summer months, when mosquitoes are most active, a timing that may have resulted in a higher proportion of owners reporting current use of HWCM. Routine use of HWCM is critically important because many of the products can limit environmental contamination with zoonotic parasites like *A. caninum* and *T. canis* which cause cutaneous *larva migrans* and toxocariasis, respectively. Other strategies such as reducing the number of stray or free-roaming animals, prompt removal of all pet feces, wearing shoes to avoid skin contact with contaminated soil, hand-washing after handling feces or soil, and avoiding geophagy in children can also reduce infection risk [48].

## Conclusions

Dog parks and other areas in which dogs are walked (e.g. neighborhood walking paths, apartment complexes) provide valuable human and animal socialization opportunities, but also may increase the risk of exposure to intestinal parasite infection. Maintaining dogs on broad-spectrum parasite control products with efficacy against hookworms, whipworms and ascarids helps mitigate this risk, decreasing the health risks to dogs and the potential for zoonotic infections, particularly as the owner-pet relationship and interaction grow ever closer. Indeed, in the present study reported use of HWCM reduced but did not eliminate infection with intestinal nematodes. Canine cestode infection prevalence remains unclear, but a recent study suggests that tapeworms are common in dogs and that routine treatment for tapeworms may also be warranted [14]. The CAI used in the present study detected more infections than did CF alone, although using the two tests in concert allowed the greatest number of infections to be identified. Regular fecal testing for parasites by CF and CAI is recommended to safeguard canine health by identifying infections early and as a means of monitoring product use and continued efficacy.

## Supplementary information

**Additional file 1: Table S1.** Number (%^a^; 95% confidence interval) of dogs visiting dog parks in each city with a positive test for intestinal parasites by coproantigen immunoassay and/or centrifugal flotation.

**Additional file 2: Table S2.** Number (%^a^; 95% confidence interval) of dog parks in each city with at least one positive fecal test (coproantigen immunoassay and/or centrifugal flotation).

## Data Availability

Data supporting the conclusions of this article are included within the article and its additional files. Raw data from this study are available for review upon reasonable request to Elanco Animal Health, IDEXX or Oklahoma State University.

## Abbreviations

CAI: coproantigen immunoassay
CF: centrifugal flotation
CI: confidence interval
FDA: Food and Drug Administration
HWCM: heartworm/intestinal parasite control medication
