# Correction to: Detection of gastrointestinal parasitism at recreational canine sites in the USA: the DOGPARCS study

**DOI:** 10.1186/s13071-020-04209-9

**Published:** 2020-07-13

**Authors:** Kristina Stafford, Todd M. Kollasch, Kathryn T. Duncan, Stephanie Horr, Troy Goddu, Christine Heinz-Loomer, Anthony J. Rumschlag, William G. Ryan, Sarah Sweet, Susan E. Little

**Affiliations:** 1 Elanco Animal Health, 2500 Innovation Way, Greenfield, IN 46140 USA; 2 Department of Veterinary Pathobiology, Center for Veterinary Health Sciences, Oklahoma State University, Stillwater, OK 74078 USA; 3 IDEXX, 1 IDEXX Dr, Westbrook, ME 04092 USA; 4 Ryan Mitchell Associates LLC, 16 Stoneleigh Park, Westfeld, NJ USA

## Correction to: Parasites Vectors (2020) 13:275 10.1186/s13071-020-04147-6

Following publication of the original article [1], the authors reported two errors: one error in the caption for Fig. 2 and one in Additional file 1: Table S1.

In the caption for Fig. 2 it said ‘…dogs < 4 years’ in place of “… dogs ≥ 4 years”.

While in Additional file 1: Table S1, it said (in the first column) ‘Boise (100)’ in place of ‘Boise (105)’.

The original article [1] has been updated to correct this.

The authors apologize for any inconvenience caused.
