# Supplementary material for: Detection of gastrointestinal parasitism at recreational canine sites in the USA: the DOGPARCS study
Source: Parasit Vectors. 2020 Jun 1;13:275. doi: 10.1186/s13071-020-04147-6 (PMC7268625; doi:10.1186/s13071-020-04147-6)
Supplement: Supplementary file 1 — Additional file 1: Table S1. Number (%a; 95% confidence interval) of dogs visiting dog parks in each city with a positive test for intestinal parasites by coproantigen immunoassay and/or centrifugal flotation. [file 13071_2020_4147_MOESM1_ESM.docx]

**Additional file 1: Table S1**. Number (%; 95% confidence interval) of dogs visiting dog parks in each city with a positive test for intestinal parasites by coproantigen immunoassay and/or centrifugal flotation

| **City (number of  dogs sampled)** | **Nematodes/*Giardia**** | **Nematodes^#^** | **Hookworms** | **Whipworms** | **Ascarids** | ***Giardia*** |
| --- | --- | --- | --- | --- | --- | --- |
| **National (N = 3006)** | **609 (20.3)** | **263 (8.8)** | **214 (7.1)** | **58 (1.9)** | **17 (0.6)** | **391 (13.0)** |
| **Southeast** |  |  |  |  |  |  |
| Atlanta (100) | 16 (16.0; 10.0–24.5) | 10 (10.0; 5.4–17.6) | 8 (8.0; 3.9–15.2) | 0 (0.0; 0.0–4.4) | 2 (2.0; 0.1–7.4) | 7 (7.0; 3.2–14.0) |
| Austin (100) | 30 (30.0; 21.9–39.6) | 13 (13.0; 7.6–21.1) | 12 (12.0; 6.9–20.0) | 2 (2.0; 0.1–7.4) | 0 (0.0; 0.0–4.4) | 20 (20.0; 13.3–29.0) |
| Charlotte (100) | 26 (26.0; 18.4–35.4) | 14 (14.0; 8.4–22.3) | 9 (9.0; 4.6–16.4) | 6 (6.0; 2.5–12.7) | 1 (1.0; 0.0–6.0) | 15 (15.0; 9.2–23.4) |
| Houston (101) | 32 (31.7; 23.4–41.3) | 23 (22.8; 15.6–31.9) | 22 (21.8; 14.8–30.9) | 2 (2.0; 0.1–7.4) | 0 (0.0; 0.0–4.4) | 10 (9.9; 5.3–17.5) |
| Miami/Ft Lauderdale (100) | 40 (40.0; 30.9–49.8) | 33 (33.0; 24.5–42.7) | 32 (32.0; 23.7–41.7) | 3 (3.0; 0.7–8.8) | 1 (1.0; 0.0–6.0) | 11 (11.0; 6.1–18.8) |
| Nashville (100) | 28 (28.0; 20.1–37.5) | 15 (15.0; 9.2–23.4) | 13 (13.0; 7.6–21.1) | 4 (4.0; 1.2–10.2) | 1 (1.0; 0.0–6.0) | 15 (15.0; 9.2–23.4) |
| New Orleans (87) | 17 (19.5; 12.5–29.2) | 10 (11.5; 6.2–20.1) | 9 (10.3; 5.3–18.7) | 1 (1.2; 0.0–6.8) | 0 (0.0; 0.0–5.1) | 8 (9.2; 4.5–17.3) |
| Oklahoma City/Tulsa (100) | 31 (31.0; 22.8–40.7) | 18 (18.0; 11.6–26.8) | 17 (17.0; 10.8–25.7) | 4 (4.0; 1.2–10.2) | 0 (0.0; 0.0–4.4) | 19 (19.0; 12.4–27.9) |
| Raleigh/Durham (100) | 22 (22.0; 14.9–31.1) | 15 (15.0; 9.2–23.4) | 13 (13.0; 7.6–21.1) | 2 (2.0; 0.1–7.4) | 0 (0.0; 0.0–4.4) | 12 (12.0; 6.9–20.0) |
| Tampa (101) | 28 (27.7; 19.9–37.2) | 18 (17.8; 11.5–26.5) | 16 (15.8; 9.9–24.3) | 3 (3.0; 0.7–8.7) | 0 (0.0; 0.0–0.4) | 12 (11.9; 6.8–19.8) |
| **Northeast** |  |  |  |  |  |  |
| Boston (100) | 17 (17.0; 10.8–25.7) | 6 (6.0; 2.5– 12.7) | 6 (6.0; 2.5–12.7) | 1 (1.0; 0.0–6.0) | 0 (0.0; 0.0–4.4) | 11 (11.0; 6.1–18.8) |
| New York City (100) | 17 (17.0; 10.8–25.7) | 5 (5.0; 1.9– 11.5) | 4 (4.0; 1.2–10.2) | 1 (1.0; 0.0–6.0) | 0 (0.0; 0.0–4.4) | 12 (12.0; 6.9–20.0) |
| Philadelphia (100) | 16 (16.0; 10.0–24.5) | 6 (6.0; 2.5– 12.7) | 5 (5.0; 1.9–11.5) | 2 (2.0; 0.1–7.4) | 0 (0.0; 0.0–4.4) | 11 (11.0; 6.1–18.8) |
| Washington DC (100) | 22 (22.0; 14.9–31.1) | 8 (8.0; 3.9– 15.2) | 6 (6.0; 2.5–12.7) | 4 (4.0; 1.2–10.2) | 1 (1.0; 0.0–6.0) | 14 (14.0; 8.4–22.3) |
| **Midwest** |  |  |  |  |  |  |
| Chicago (100) | 24 (24.0; 16.6–33.3) | 4 (4.0; 1.2–10.3) | 2 (2.0; 0.1–7.4) | 1 (1.0; 0.0–6.0) | 1 (1.0; 0.0–6.0) | 22 (22.0; 14.9–31.1) |
| Cleveland (101) | 23 (22.8; 15.6–31.9) | 11 (10.9; 6.0–18.6) | 4 (4.0; 1.2–10.1) | 5 (5.0; 1.9–11.4) | 4 (4.0; 1.2–10.1) | 20 (19.8; 13.1–28.7) |
| Detroit (106) | 12 (11.3; 6.5–18.9) | 4 (3.8; 1.2–9.6) | 4 (3.8; 1.2–9.6) | 0 (0.0; 0.0–4.2) | 0 (0.0; 0.0–4.2) | 9 (8.5; 4.4–15.5) |
| Indianapolis (101) | 12 (11.9; 6.8–19.8) | 4 (4.0; 1.2–10.1) | 3 (3.0; 0.7–8.7) | 2 (2.0; 0.1–7.4) | 0 (0.0; 0.0–4.4) | 8 (7.9; 3.9–15.1) |
| Kansas City (100) | 17 (17.0; 10.8–25.7) | 6 (6.0; 2.5–12.7) | 5 (5.0; 1.9–11.5) | 1 (1.0; 0.0–6.0) | 0 (0.0; 0.0–4.4) | 11 (11.0; 6.1–18.8) |
| Minneapolis (100) | 19 (19.0; 12.4–27.9) | 8 (8.0; 3.9–15.2) | 7 (7.0; 3.2–14.0) | 2 (2.0; 0.1–7.4) | 1 (1.0; 0.0–6.0) | 11 (11.0; 6.1–18.8) |
| St Louis (100) | 24 (24.0; 16.6–33.3) | 7 (7.0; 3.2–14.0) | 3 (3.0; 0.7–8.8) | 4 (4.0; 1.2–10.2) | 0 (0.0; 0.0–4.4) | 17 (17.0; 10.8–25.7) |
| **West** |  |  |  |  |  |  |
| Albuquerque (102) | 12 (11.8; 6.7–19.6) | 3 (2.9; 0.6–8.7) | 3 (2.9; 0.6–8.7) | 2 (2.0; 0.1–7.3) | 0 (0.0; 0.0–4.4) | 9 (8.8; 4.5–16.1)) |
| Bakersfield (100) | 15 (15.0; 9.2–23.4) | 5 (5.0; l 1.9–11.5) | 0 (0.0; 0.0–4.4) | 3 (3.0; 0.7–8.8) | 2 (2.0; 0.1–7.4) | 12 (12.0; 6.9–20.0) |
| Boise (100) | 13 (12.4; 7.3–20.2) | 1 (1.0; 0.0–5.7) | 1 (1.0; 0.0–5.7) | 0 (0.0; 0.0–4.2) | 0 (0.0; 0.0–4.2) | 12 (11.4; 6.5–19.1) |
| Denver (103) | 11 (10.7; 5.9–18.3) | 2 (1.9; 0.1–7.2) | 1 (1.0; 0.0–5.8) | 0 (0.0; 0.0–4.3) | 1 (1.0; 0.0–5.8) | 10 (9.7; 5.2–17.1) |
| Los Angeles (100) | 12 (12.0; 6.9–20.0) | 0 (0.0; 0.0–4.4) | 0 (0.0; 0.0–4.4) | 0 (0.0; 0.0–4.4) | 0 (0.0; 0.0–4.4) | 12 (12.0; 6.9–20.0) |
| Phoenix (100) | 23 (23.0; 15.8–32.2) | 6 (6.0; 2.5–12.7) | 5 (5.0; l 1.9–11.5) | 1 (1.0; 0.0–6.0) | 0 (0.0; 0.0–4.4) | 18 (18.0; 11.6–26.8) |
| Portland (100) | 19 (19.0; 12.4–27.9) | 1 (1.0; 0.0–6.0) | 1 (1.0; 0.0–6.0) | 0 (0.0; 0.0–4.4) | 0 (0.0; 0.0–4.4) | 18 (18.0; 11.6–26.8) |
| Sacramento (99) | 15 (15.2; 9.3–23.6) | 3 (3.0; 0.7–8.9) | 1 (1.0; 0.0–6.1) | 1 (1.0; 0.0–6.1) | 1 (1.0; 0.0–6.1) | 12 (12.1; 6.9–20.2) |
| Seattle (100) | 16 (16.0; 10.0–24.5) | 4 (4.0; 1.2–10.2) | 2 (2.0; 0.1–7.4) | 1 (1.0; 0.0–6.0) | 1 (1.0; 0.0–6.0) | 13 (13.0; 7.6–21.1) |

*Note*: Percentages based on the number of positive dogs in a city as the numerator and total of dogs sampled in that city as denominator;

**^*^**Includes species of hookworms– whipworms– ascarids– and *Giardia*; **^#^**Includes species of hookworms– whipworms and ascarids
