# Supplementary material for: Detection of gastrointestinal parasitism at recreational canine sites in the USA: the DOGPARCS study
Source: Parasit Vectors. 2020 Jun 1;13:275. doi: 10.1186/s13071-020-04147-6 (PMC7268625; doi:10.1186/s13071-020-04147-6)
Supplement: Supplementary file 2 — Additional file 2: Table S2. Number (%a; 95% confidence interval) of dog parks in each city with at least one positive fecal test (coproantigen immunoassay and/or centrifugal flotation). [file 13071_2020_4147_MOESM2_ESM.docx]

**Additional file 2: Table S2.** Number (%; 95% confidence interval) of dog parks in each city with at least one positive fecal test (coproantigen immunoassay and/or centrifugal flotation)

| **City (number of  parks sampled)** | **Nematodes/*Giardia*^*^** | **Nematodes^#^** | **Hookworms** | **Whipworms** | **Ascarids** | ***Giardia*** |
| --- | --- | --- | --- | --- | --- | --- |
| **National (N = 288)** | **243 (84.4)** | **143 (49.7)** | **125 (43.4)** | **52 (18.1)** | **16 (5.6)** | **213 (74.0)** |
| **Southeast** |  |  |  |  |  |  |
| Atlanta (10) | 7 (70.0; 39.2–89.7) | 6 (60.0; 31.2–83.3) | 5 (50.0; 23.7–76.3) | 0 (0.0; 0.0–32.1) | 2 (20.0; 4.6–52.1) | 5 (50.0; 23.7–76.3) |
| Austin (10) | 9 (90.0; 57.4–100.0) | 8 (80.0; 47.9–95.4) | 8 (80.0; 47.9–95.4) | 2 (20.0; 4.6–52.1) | 0 (0.0; 0.0–32.1) | 9 (90.0; 57.4–100.0) |
| Charlotte (10) | 10 (100; 67.9–100.0) | 7 (70.0; 39.2–89.7) | 6 (60.0; 31.2–83.3) | 5 (50.0; 23.7–76.3) | 1 (10.0; 0.0–42.6) | 9 (90.0; 57.4–100.0) |
| Houston (9) | 8 (88.9; 54.3–100.0) | 7 (77.8; 44.3–94.7) | 7 (77.8; 44.3–94.7) | 2 (22.2; 5.3–55.7) | 0 (0.0; 0.0–34.5) | 6 (66.7; 35.1–88.3) |
| Miami/Ft Lauderdale (10) | 10 (100; 67.9–100.0) | 10 (100; 67.9–100.0) | 10 (100; 67.9–100.0) | 3 (30.0; 10.3–60.8) | 1 (10.0; 0.0–42.6) | 7 (70.0; 39.2–89.7) |
| Nashville (8) | 7 (87.5; 50.8–99.9) | 6 (75.0; 40.1–93.7) | 6 (75.0; 40.1–93.7) | 4 (50.0; 21.5–78.5) | 1 (12.5; 0.1–49.2) | 6 (75.0; 40.1–93.7) |
| New Orleans (9) | 7 (77.8; 44.3–94.7) | 4 (44.4; 18.8–73.4) | 3 (33.3; 11.7–64.9) | 1 (11.1; 0.0–45.7) | 0 (0.0; 0.0–34.5) | 4 (44.4; 18.8–73.4) |
| Oklahoma City/Tulsa (10) | 10 (100; 67.9–100.0) | 9 (90.0; 57.4–100.0) | 8 (80.0; 47.9–95.4) | 4 (40.0; 16.7–68.8) | 0 (0.0; 0.0–32.1) | 9 (90.0; 57.4–100.0) |
| Raleigh/Durham (10) | 9 (90.0; 57.4–100.0) | 7 (70.0; 39.2–89.7) | 7 (70.0; 39.2–89.7) | 2 (20.0; 4.6–52.1) | 0 (0.0; 0.0–32.1) | 8 (80.0; 47.9–95.4) |
| Tampa (10) | 9 (90.0; 57.4–100.0) | 9 (90.0; 57.4–100.0) | 9 (90.0; 57.4–100.0) | 2 (20.0; 4.6–52.1) | 0 (0.0; 0.0–32.1) | 7 (70.0; 39.2–89.7) |
| **Northeast** |  |  |  |  |  |  |
| Boston (9) | 8 (88.9; 54.3–100.0) | 5 (55.6; 26.6–81.2) | 5 (55.6; 26.6–81.2) | 1 (11.1; 0.0–45.7) | 0 (0.0; 0.0–34.5) | 6 (66.7; 35.1–88.3) |
| New York City (10) | 7 (70.0; 39.2–89.7) | 3 (30.0; 10.3–60.8) | 2 (20.0; 4.6–52.1) | 1 (10.0; 0.0–42.6) | 0 (0.0; 0.0–32.1) | 7 (70.0; 39.2–89.7) |
| Philadelphia (10) | 8 (80.0; 47.9–95.4) | 4 (40.0; 16.7–68.8) | 4 (40.0; 16.7–68.8) | 1 (10.0; 0.0–42.6) | 0 (0.0; 0.0–32.1) | 8 (80.0; 47.9–95.4) |
| Washington DC (n = 10) | 8 (80.0; 47.9–95.4) | 5 (50.0; 23.7–76.3) | 5 (50.0; 23.7–76.3) | 4 (40.0; 16.7–68.8) | 1 (10.0; 0.0–42.6) | 7 (70.0; 39.2–89.7) |
| **Midwest** |  |  |  |  |  |  |
| Chicago (10) | 9 (90.0; 57.4–100.0) | 4 (40.0; 16.7–68.8) | 2 (20.0; 4.6–52.1) | 1 (10.0; 0.0–42.6) | 1 (10.0; 0.0–42.6) | 9 (90.0; 57.4–100.0) |
| Cleveland (9) | 8 (88.9; 54.3–100.0) | 5 (55.6; 26.6–81.2) | 3 (33.3; 11.7–64.9) | 4 (44.4; 18.8–73.4) | 3 (33.3; 11.7–64.9) | 7 (77.8; 44.3–94.7) |
| Detroit (10) | 8 (80.0; 47.9–95.4) | 4 (40.0; 16.7–68.8) | 4 (40.0; 16.7–68.8) | 0 (0.0; 0.0–32.1) | 0 (0.0; 0.0–32.1) | 7 (70.0; 39.2–89.7) |
| Indianapolis (9) | 6 (66.7; 35.1–88.3) | 3 (33.3; 11.7–64.9) | 3 (33.3; 11.7–64.9) | 1 (11.1; 0.0–45.7) | 0 (0.0; 0.0–34.5) | 5 (55.6; 26.6–81.2) |
| Kansas City (10) | 9 (90.0; 57.4–100.0) | 4 (40.0; 16.7–68.8) | 4 (40.0; 16.7–68.8) | 1 (10.0; 0.0–42.6) | 0 (0.0; 0.0–32.1) | 6 (60.0; 31.2–83.3) |
| Minneapolis (10) | 10 (100; 67.9–100.0) | 7 (70.0; 39.2–89.7) | 7 (70.0; 39.2–89.7) | 2 (20.0; 4.6–52.1) | 1 (10.0; 0.0–42.6) | 7 (70.0; 39.2–89.7) |
| St Louis (10) | 9 (90.0; 57.4–100.0) | 6 (60.0; 31.2–83.3) | 3 (30.0; 10.3–60.8) | 3 (30.0; 10.3–60.8) | 0 (0.0; 0.0–32.1) | 9 (90.0; 57.4–100.0) |
| **West** |  |  |  |  |  |  |
| Albuquerque (10) | 7 (70.0; 39.2–89.7) | 3 (30.0; 10.3–60.8) | 3 (30.0; 10.3–60.8) | 2 (20.0; 4.6–52.0) | 0 (0.0; 0.0–32.1) | 7 (70.0; 39.2–89.7) |
| Bakersfield (9) | 7 (77.8; 44.3–94.7) | 4 (44.3; 18.8–73.4) | 0 (0.0; 0.0–34.5) | 3 (33.3; 11.7–64.9) | 2 (22.2; 5.3–55.7) | 7 (77.8; 44.3–94.7) |
| Boise (10) | 6 (60.0; 31.2–83.3) | 1 (10.0; 0.0–42.6) | 1 (10.0; 0.0–42.6) | 0 (0.0; 0.0–32.1) | 0 (0.0; 0.0–32.1 | 6 (60.0; 31.2–83.3) |
| Denver (10) | 7 (70.0; 39.2–89.7) | 2 (20.0; 4.6–52.1) | 1 (10.0; 0.0–42.6) | 0 (0.0; 0.0–32.1) | 1 (10.0; 0.0–42.6) | 7 (70.0; 39.2–89.7) |
| Los Angeles (9) | 7 (77.8; 44.3–94.7) | 0 (0.0; 0.0–34.5) | 0 (0.0; 0.0–34.5) | 0 (0.0; 0.0–34.5) | 0 (0.0; 0.0–34.5) | 7 (77.8; 44.3–94.7) |
| Phoenix (10) | 10 (100; 67.9–100.0) | 5 (50.0; 23.7–76.3) | 5 (50.0; 23.7–76.3) | 1 (10.0; 0.0–42.6) | 0 (0.0; 0.0–32.1) | 10 (100; 67.9–100.0) |
| Portland (10) | 8 (80.0; 47.9–95.4) | 1 (10.0; 0.0–42.6) | 1 (10.0; 0.0–42.6) | 0 (0.0; 0.0–32.1) | 0 (0.0; 0.0–32.1) | 8 (80.0; 47.9–95.4) |
| Sacramento (7) | 7 (100; 59.6–100.0) | 1 (14.3; 0.5–53.4) | 1 (14.3; 0.5–53.4) | 1 (14.3; 0.5–53.4) | 1 (14.3; 0.5–53.4) | 6 (85.7; 46.7–99.5) |
| Seattle (10) | 8 (80.0; 47.9–95.4) | 3 (30.0; 10.3–60.8) | 2 (20.0; 4.6–52.1) | 1 (10.0; 0.0–42.6) | 1 (10.0; 0.0–42.6) | 7 (70.0; 39.2–89.7) |

*Note*: Percentages based on the number of positive dog parks in a city as the numerator and total of dog parks sampled in that city as denominator;

**^*^**Includes species of hookworms–whipworms–ascarids–and *Giardia*; **^#^**Includes species of hookworms–whipworms and ascarids
